# False positive FDG uptake in melanoma patients treated with talimogene laherparepvec (T‐VEC)

**DOI:** 10.1002/jso.26607

**Published:** 2021-07-08

**Authors:** Evalyn E. A. P. Mulder, Emma H. A. Stahlie, Daniëlle Verver, Clara Lemstra, Lukas B. Been, Antien L. Mooyaart, Tessa Brabander, Erik Vegt, Frederik A. Verburg, Astrid A. M. van der Veldt, Cornelis Verhoef, Alexander C. J. van Akkooi, Dirk J. Grünhagen

**Affiliations:** ^1^ Department of Surgical Oncology Erasmus MC Cancer Institute Rotterdam The Netherlands; ^2^ Department of Medical Oncology Erasmus MC Cancer Institute Rotterdam The Netherlands; ^3^ Department of Surgical Oncology Netherlands Cancer Institute – Antoni van Leeuwenhoek Amsterdam The Netherlands; ^4^ Department of Surgical Oncology University Medical Center Groningen Groningen The Netherlands; ^5^ Department of Pathology Erasmus MC Rotterdam The Netherlands; ^6^ Department of Radiology & Nuclear Medicine Erasmus MC Cancer Institute Rotterdam The Netherlands

**Keywords:** cutaneous melanoma, false positive FDG uptake, FDG‐PET/CT, talimogene laherparevec (T‐VEC)

## Abstract

Talimogene laherparepvec (T‐VEC) is a genetically modified herpes simplex virus‐1‐based oncolytic immunotherapy and has been approved for the local treatment of unresectable (stage IIIB/C and IVM1a) cutaneous melanoma. During T‐VEC treatment, tumor response is often evaluated using [18F]2‐fluoro‐2‐deoxy‐ d‐glucose(FDG) positron emission tomography/computed tomography (PET/CT). In a Dutch cohort (*n* = 173), almost one‐third of patients developed new‐onset FDG uptake in uninjected locoregional lymph nodes during T‐VEC. In 36 out of 53 (68%) patients with new nodal FDG uptake, nuclear medicine physicians classified this FDG uptake as “suspected metastases” without clinical or pathological confirmation in the majority of patients. These false positive results indicate that new‐onset FDG uptake in locoregional lymph nodes during T‐VEC treatment does not necessarily reflect progressive disease, but may be associated with immune infiltration. In current clinical practice, physicians should be aware of the high false positive rate of FDG uptake during treatment with T‐VEC in patients with melanoma. Therefore, pathological examination of lymph node lesions with new FDG uptake is recommended to differentiate between progressive disease and immune infiltration after treatment with T‐VEC.

## Highlights

- FDG‐PET/CT is commonly used for response evaluation of T‐VEC in melanoma patients.
- New‐onset FDG uptake in uninjected locoregional lymph nodes occurs frequently.
- New FDG uptake can reflect progressive disease or immune infiltration.
- Pathological examination of the new‐onset FDG avid lymph node is recommended.

## 1 INTRODUCTION

Talimogene laherparepvec (T‐VEC) is an oncolytic immunotherapy and has been approved for local treatment of unresectable (stage IIIB/C and IVM1a) cutaneous melanoma.^1^, ^2^ After intralesional injection in (sub)cutaneous and/or nodal lesions, the genetically modified herpes simplex virus‐1 selectively replicates in tumor cells and produces granulocyte‐macrophage colony‐stimulating factor.^3^, ^4^ T‐VEC promotes lysis and cell death, thereby inducing an antitumor immune response, which is local but can also be systemic.^5^, ^6^ Since the introduction of T‐VEC in the clinic, durable tumor responses have been described,^7^ and best overall response rates have been reported up to 89%.^8^ In addition to T‐VEC, immune checkpoint inhibitors and targeted therapy have proven efficacy and have been approved for the treatment of unresectable and metastatic melanoma.^9^, ^10^, ^11^

To adequately select patients for T‐VEC, [18F]2‐fluoro‐2‐deoxy‐d‐glucose (FDG) positron emission tomography/computed tomography (PET/CT) is usually performed to identify patients without distant metastases. As patients with unresectable stage III and IVM1a melanoma are at high risk of distant metastases, adequate response evaluation is essential to switch to other treatments in case of progressive disease during T‐VEC. Since FDG‐PET/CT has a high sensitivity for advanced melanoma,^12^, ^13^ it is often applied for early detection of (distant) metastasis during treatment with T‐VEC.^14^ In addition, FDG‐PET/CT can guide the discontinuation of T‐VEC since the complete metabolic response on FDG‐PET/CT is indicative of a complete pathological response during T‐VEC.^14^ On the other hand, the development of new FDG avid lesions suggests progressive disease, requiring another treatment strategy. However, tumor responses during immunomodulatory treatments are associated with development of immune infiltrates, which can also cause increased FDG uptake.^12^, ^13^ False positive FDG uptake during T‐VEC may result in treatment discontinuation. To evaluate this diagnostic dilemma in clinical practice, a study was performed to evaluate the false positive FDG uptake in locoregional lymph nodes in patients treated with T‐VEC in three dedicated melanoma centers in The Netherlands.

## 2 MATERIALS AND METHODS

Consecutive patients treated with T‐VEC were retrospectively selected in one of the following three Dutch melanoma centers: Netherlands Cancer Institute – Antoni van Leeuwenhoek, Erasmus MC Cancer Institute, and University Medical Center Groningen. In these centers, FDG‐PET with (low dose) CT is performed every 3–6 months according to clinical practice during treatment with T‐VEC. To limit detection of FDG uptake in response to T‐VEC, FDG‐PET/CT is preferably scheduled at 1–2 weeks after the last administration of T‐VEC. In addition, visible and/or palpable lesions are usually evaluated clinically every 2 weeks during T‐VEC treatment.

For this analysis, patients without distant metastases on FDG‐PET/CT before initiation of T‐VEC treatment were included. At baseline and after T‐VEC treatment, the clinical reports of FDG‐PET/CT, routinely made by experienced nuclear medicine physicians, were reviewed for new‐onset FDG uptake in uninjected locoregional lymph nodes and changes in nodal size (short axis, in mm). New‐onset FDG uptake was described by the nuclear medicine physicians as either “suspected metastases,” “probably reactive,” or “equivocal” (malignant or reactive). Pathological examination was performed to confirm progressive disease in lymph nodes with new‐onset FDG uptake. Tissue was obtained through (core needle) biopsy, fine needle aspiration, or surgical excision. When pathological examination was not feasible or inconclusive, true lesion status of new‐onset FDG uptake in locoregional lymph nodes was determined by routine follow‐up, including physical examination and FDG‐PET/CT. New‐onset FDG uptake in uninjected locoregional lymph nodes was classified as false positive if pathological examination did not demonstrate malignant cells and/or FDG uptake disappeared spontaneously during follow‐up.

## 3 RESULTS

**Figure 1:**
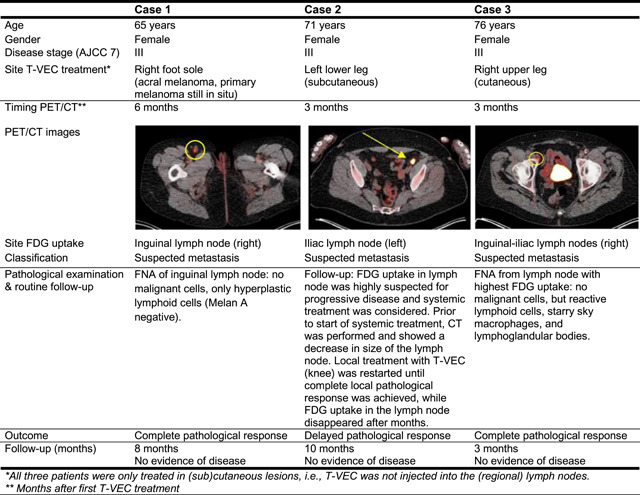
False positive FDG uptake in locoregional lymph nodes in three patients treated with T‐VEC. AJCC, American Joint Committee on Cancer; CT, computed tomography; FDG‐PET, [18F]2‐fluoro‐2‐deoxy‐d‐glucose positron emission tomography; FNA, fine needle aspiration; T‐VEC, talimogene laherparepvec [Color figure can be viewed at wileyonlinelibrary.com]

Between December 2016 and August 2020, 238 patients with melanoma were treated with T‐VEC and 173 patients underwent FDG‐PET/CT at baseline and during treatment with T‐VEC. In these 173 included patients, the median number of injected lesions was 4 (interquartile range [IQR] = 2–10) with a median amount of 2 ml per T‐VEC treatment (IQR = 1–2 ml). After 3–12 months of T‐VEC treatment, 53 out of 173 (31%) patients had new FDG uptake in uninjected locoregional lymph nodes nearby T‐VEC injected (sub)cutaneous lesions. In the majority of these patients (*n* = 42, 79%), FDG uptake was not associated with malignancy (Table 1), but with immune infiltration. For new‐onset FDG uptake, median nodal size was comparable between malignant (8.2 mm [IQR = 7.7–10.1 mm]) and nonmalignant (9.9 mm [IQR = 8.0–11.0 mm]) FDG avid lesions. In 36 out of 53 patients (68%) with new‐onset FDG uptake in locoregional lymph nodes, lesions were classified as “suspected metastases,” which was not confirmed by pathological examination or routine follow‐up (median 18 months [IQR = 11–25 months]) in the majority of patients (27 out of 36 patients, 75%). For illustrative purposes, three patients with false positive FDG uptake in locoregional lymph nodes are shown in Figure 1.

**Table 1:** Correlation of new‐onset FDG uptake in locoregional lymph nodes in melanoma patients treated with T‐VEC

	Patients with new nodal FDG uptake	Classification of new nodal FDG uptake		
		Suspected metastases	Probably reactive	Equivocal
	*n* = 53	*n* = 36	*n* = 10	*n* = 7
**True positive** (malignant)	**11** (21%)	**9** (25%)	**1** (10%)	**1** (14%)
Pathology	7			
Follow‐up	4			
**False positive** (nonmalignant)	**42** (79%)	**27** (75%)	**9** (90%)	**6** (86%)
Pathology	24			
Follow‐up	18			

*Note*: FDG‐PET/CT evaluation was performed in 173 patients. After 3–12 months of T‐VEC treatment, 53 (31%) patients had new FDG uptake in uninjected locoregional lymph nodes nearby T‐VEC injected (sub)cutaneous lesions.

Abbreviations: FDG, [18F]2‐fluoro‐2‐deoxy‐d‐glucose positron emission tomography; PET/CT, positron emission tomography/computed tomography; T‐VEC, talimogene laherparepvec.

## 4 DISCUSSION

This Dutch cohort shows that almost one‐third of patients developed new‐onset FDG uptake in uninjected locoregional lymph nodes during T‐VEC, without pathological and/or clinical confirmation of progressive disease in the majority of patients. At pathological examination, false positive FDG uptake was associated with T‐VEC‐induced immune infiltration. In patients with advanced‐stage melanoma, comparable observations have been described after isolated limb perfusion and treatment with immune checkpoint inhibitors.^15^, ^16^, ^17^ Immunomodulatory therapies, including T‐VEC, boost the immune system to enhance an effective T‐cell mediated antitumor response.^18^ Our findings indicate that new‐onset FDG uptake in locoregional lymph nodes during T‐VEC treatment occurs frequently and does not necessarily reflect progressive disease. According to the response evaluation criteria in solid tumors (RECIST) v1.1,^19^ lymph nodes < 10 mm are considered benign. However, in our cohort, the vast majority of the FDG avid lymph nodes, both benign and malignant, were <10 mm. Therefore, RECIST cannot contribute to further characterization of FDG avid nodal lesions after T‐VEC. Pathological examination of lymph node lesions with new FDG uptake is required to differentiate between progressive disease and immune infiltration before (dis)continuation of T‐VEC and/or switch to other therapy. Prospective studies are needed to determine the diagnostic accuracy and clinical value of FDG‐PET/CT for tumor response evaluation during treatment with T‐VEC in melanoma patients.

## 5 CONCLUSION

In current clinical practice, physicians should be aware of the high rate of false positive FDG uptake in locoregional lymph nodes after treatment with T‐VEC in patients with unresectable melanoma. To address this issue, it is recommended to obtain representative tissue.

## CONFLICT OF INTERESTS

Astrid A.M. van der Veldt: Consultant and/or advisory board member BMS, MSD, Merck, Ipsen, Eisai, Pfizer, Novartis, Pierre Fabre, Sanofi, and Roche. All paid to institute and unrelated to current work. Lukas B. Been: Advisory board member BMS. All paid to institute and unrelated to current work. Alexander C.J. van Akkooi: Consultant and/or advisory board member: Amgen, Bristol‐Myers Squibb, Novartis, MSD Merck, Merck‐Pfizer, Sanofi, Sirius Medical, 4SC. Research grants: Amgen, Merck‐Pfizer. All paid to institute and unrelated to current work. Evalyn E. A. P. Mulder, Emma H. A. Stahlie, Daniëlle Verver, Clara Lemstra, Antien L. Mooyaart, Tessa Brabander, Erik Vegt, Frederik A. Verburg, Cornelis Verhoef, and Dirk J. Grünhagen have declared no competing interests.

## ETHICS STATEMENT

This study has been approved by the Erasmus Medical Center Ethics Committee and was conducted according to the principles of the Declaration of Helsinki (10th version, Fortaleza 2013) and in concordance with the Dutch Medical Research Improving Human Subjects Act. In view of the retrospective nature of the study and as procedures performed were part of the routine care, the Ethics Committee waived the need for written informed consent.

## SYNOPSIS

Talimogene laherparepvec (T‐VEC) is a genetically modified herpes simplex virus‐1‐based oncolytic immunotherapy and has been approved for the local treatment of unresectable (stage IIIB/C and IVM1a) cutaneous melanoma. During T‐VEC treatment, tumor response is often evaluated using [18F]2‐fluoro‐2‐deoxy‐d‐glucose (FDG) positron emission tomography/computed tomography (PET/CT). Our findings indicate that new‐onset FDG uptake in uninjected locoregional lymph nodes in patients treated with T‐VEC occurs frequently and does not necessarily reflect progressive disease, but may be associated with immune infiltration; to address this issue, it is recommended to obtain representative tissue.

## Data Availability

The data that support the findings of this study are available from the corresponding author upon reasonable request.
